# Objects of the Organization

**Published:** 1902-02

**Authors:** R. R. Kime

**Affiliations:** Atlanta, Ga., President Georgia State Sociologicol Society, ex-President Tri-State Medical Society, ex-President Atlanta Medical Society, member American Medical Association, Southern Surgical and Gynecological, Tri-State and Georgia State Medical Societies


### OBJECTS OF THE ORGANIZATION.*

BY R. R. KIME, M.D., ATLANTA, GA.,

President Georgia State Sociologicol Society, ex-President Tri-State Medical Society, ex-President Atlanta Medical Society, member American Medical Association, Southern Surgical and Gynecological, Tri-State and Georgia State Medical Societies.

In considering the questions of crime, vice and disease, with their serious results to the welfare of the human race, we are impressed with the necessity of instituting measures to prevent their development and results.

While there are a vast number and varied influences operating to curtail, suppress and control these powerful influences that deteriorate and degrade the race, there are yet other lines of work and a great amount of good to be accomplished.

The profession of medicine during the 19th century has taught us practical lessons of immense value in the prevention of disease, which is only a beginning of the benefits to be derived if the general public will only cooperate and utilize the means at their command. If the same principles are applied in the prevention of crime and vice, equally as beneficial results to mankind may be attained.

In preventive medicine, the primal course or origin of disease, with its mode of propagation and dissemination, must be studied in order to attain success. So, if we desire to succeed in preventing crime and vice, we must study to ascertain the primal causes or origin, their mode of propagation and dissemination rather than dealing with the results or effects of preexisting forces.

The criminal is but the result of preexisting causes, and in some instances his destiny is sealed and determined before his birth, others by environment and training after birth.

Then in the name of justice is it right for us to punish the criminal regardless of his origin and incentive to crime, when we stand idly by with folded hands and do nothing to prevent his development, and even in some instances give sanction and license for the very things that produce criminals?

It has been said, "The criminal is a microbe which only flour-

---

*Read at meeting Georgia State Sociological Society, Dec. 13, 1901, Atlanta, Ga.

ishes in a suitable soil"; also, "Societies have the criminals they deserve."

These are heavy indictments against every community where crime exists. We should take an impassionate, considerate, serious view of this question when men who have made a study of these questions tell us that crime, insanity and degeneracy are on the increase.

When we think over this matter seriously, reasoning from cause to effect, at the same time recognizing our obligations to each other and to humanity, we are forced to the conclusion that you and I are in a measure directly or indirectly responsible for the crime and degeneracy that exists in our community; that each community and State are what its citizens collectively make it. If murder, theft, vice and immorality exist in Atlanta and State of Georgia, who is responsible except her citizens?

Responsible by permission or commission. Responsible by indifference, apathy, inaction and unconcern about the things which so vitally concern us as a community, state and nation.

Evil influences, vice, crime, disease and degeneracy, are ever present with us; their forces are continually at work, their effect constantly witnessed every day in our midst. To counteract these conditions equally as effective forces and measures must be instituted, not transient, spasmodic, isolated efforts, but combined, well organized persistent work.

To wage a successful warfare against these combined forces for evil will require time, study and investigation as to the cause and dissemination of each.

Good or evil is developed, not by chance or luck, but on well recognized principles and very frequently from small beginnings. It is those primary, at times seemingly insignificant, little things, that we must consider in successful sociological work. It is the tiny microscopical germ that plays havoc in medicine, producing devastation and destruction in many instances. Epidemics of small-pox, diphtheria, scarlet fever, cholera, yellow fever, etc., are due to their respective individual tiny organism. Prevent their entrance and development, then you prevent the disease and epidemic.

So it is with all septic conditions, and especially in that trying ordeal of the crowning function of motherhood, where in the past so many lives have been wrecked. But to-day we give thanks to preventive medicine for the great good accomplished in these cases by strict cleanliness, asepsis and antiseptic methods, which are preventive and destructive to germ life.

While these deal mostly with the physical side of life and disease of the physical, there are higher and greater realms yet to be explored, invaded and conquered. In the moral, mental and spiritual of mankind there are germs of vice and diseases equally as destructive as in the physical.

To eradicate these we must go hand in hand with the physical if we expect successful work, as they are interlinked, one interdependent on the other and all dependent upon the physical.

Success will be achieved much quicker by combining our forces on one common field of work where the physician, lawyer, minister, educator, journalist, business man and laborer, may join hands in advancing the common interest of humanity.

It is the object of this organization to unite these various interests in the study of crime, vice and disease; to ascertain the cause in each case and institute measures of prevention. It is easier and far less expensive, to say nothing of the loss of life, to prevent an epidemic of small-pox, diphtheria, yellow-fever, etc., than to control an epidemic after it is under full headway; so it is with crime, vice and immorality. A boy does not develop into a full grown man, a girl into a woman, in a day; a shrub into a tree at one bound, neither is a criminal or a thief developed in a day.

The criminal and thief are but the result of previous hereditary forces and the soil in which they are developed.

I would that we could free ourselves from the responsibilities of the existence of evil; that we cannot do until we have used all the means at our command to prevent, suppress and eradicate crime, vice and disease. With this object in view we are assembled to-night, and may this hour mark the beginning of a powerful organization for good, the influence of which may reach beyond the confines of our own State and bless humanity at large.

It is not designed that we should confine ourselves to one line of thought or work, but that as we progress we may investigate many questions that pertain to the higher and purer social life of man.

To do this successfully we must have committees to pursue special lines of work and report to this society from year to year the results of their investigations, with suggestions as to the best methods of preventing or correcting the evils with which they deal. We desire to direct attention briefly to some of the more important subjects that occur to the mind of the writer as worthy of committee work.

1. Crime and its Prevention, including a study of the criminal and his development.

This committee should consider the different types of criminals, gather statistics to demonstrate the effects of heredity, environment, training, alcoholism, etc., have in the development of the criminal. After doing this, then suggest methods of removing cause and preventing the development of the criminal.

The committee should also consider the value of indeterminate sentences and a system of rewards, in the reformation and control of the criminal.

2. Insanity, including Mental Degeneracy. This subject should have systematic study as to primal causes or factors, with properly selected and collected statistics to demonstrate the relation of cause and effect, considering the relation of marriage, heredity and alcoholism.

3. Tuberculosis. This is a subject of vast importance at the present time, when it sacrifices so many lives and the disease is on the increase. This committee should, after careful investigation, suggest the best means of educating the public in the prevention of tuberculosis, also the relation of the boards of health and public in the control and care of tuberculous persons.

4. Education, Public Schools, and Child Labor. These subjects are best combined, as they are closely related.

To prohibit child labor in factories and mills without providing or requiring proper training and education is substituting one evil for another. If they are left to run at large upon the streets, without education, or employed at other work, for longer time, equally as injurious and for less pay, then they are not improved physically, morally, or intellectually.

We need compulsory education, not only for factory children, but all others, with a just and equable settlement of the labor question, considering both humanitarian and business interests.

Our present methods of education are defective in many respects, and the common school system not as effective as it should be for efficient work. Here it is we need the combined influence, experience and knowledge of all persons interested, to study and discuss this subject, developing a public sentiment in favor of the most efficient methods of education that will accomplish the most good.

5. Alcoholism is a subject of vast importance and demands careful scientific, statistical, accurate study, divorcing it from politics, sectionalism and radicalism. It should be considered and investigated from two standpoints. 1st, as to its cause, development and prevention. 2d, its relation as a causative factor in the production of crime, insanity, mental and physical degeneracy.

6. Diseases and Sanitary Science. A committee under this heading could find a wide field of work for social improvement. It could show the necessity for and aid in securing a State Board of Health with systematic organization throughout the State, aid the various local boards, help educate the public in the prevention of disease, and consider the best methods of controlling the social vice and the diseases resulting therefrom. The social vice is one of the most difficult and delicate subjects we have to handle, being so intimately intermingled and woven into our social fabric. To handle it successfully will require rare discrimination and judgment, with proper education of the public as to its development, dissemination and blighting influence. This committee should see that proper textbooks on the physiological functions of the reproductive organs are written to be used for instruction of boys and girls in our high schools and academies.

7. The Negro Problem. A question of immense importance to this section of the country, not from a racial standpoint, but from a moral, intellectual, physical, sanitary and financial point of view. Separation and colonization is the most rational, practical solution of the question. Being unable at present to accomplish this, we must deal with this question as it exists and try to alleviate some of the present evils and obviate future difficulties that are sure to arise unless prevented by judicious study, investigation and handling of this subject. Will the race become extinct? If so, how and by what means? In becoming so what influence will it have upon the white race? These are burning and live questions of this day and hour.

We cannot submit to equalization or supremacy and sustain our manhood and self-respect as a people. We must face this question, we cannot evade it. The result is inevitable, and will be what we make it, whether by action or inaction. Shall we by inaction and indifference let this question settle itself by lapse of time?

The peace and purity of our homes cry, No! Shall we profit by the experience of the last thirty years on this question? No Southern home, at least in the rural sections, is safe from the "black fiend." Already many families are leaving the rural sections for better protection, which will seriously affect our agricultural interests. As a class they are unsanitary, morally and physically, forming hotbeds for the development of contagious and infectious diseases, which are a constant menace to the general public. Thus consumption, venereal diseases and contagious diseases are often disseminated.

They have equally good, and in some cases better, facilities for education than the white race, and what the result?

Let this committee study this question of the negro and his future; considering the forces that tend to his degeneration and their influence upon the whole race, giving us the unvarnished facts that we may profit thereby.

8. A committee on Publication, Lectures, and Distribution of Literature, will be an essential factor in the dissemination of such knowledge as the various committees may consider essential for the advancement of the work. Lecture courses and printed matter, judiciously handled, would aid materially in the work of this society.

9. A committee on Legislation and Uniform Laws, will be of practical value in securing judicious and proper legislation on social questions. This committee should also try to secure uniform laws in the different States on all questions of common interest to each State.

We have endeavored to briefly outline, in part at least, what we hope will be the object and scope of this organization. We trust that in the forming of this society every effort will be made to secure harmony and uniformity of action, avoiding all sectarian and political alliances that so often mar the usefulness of otherwise good organizations.

May this society be composed of a band of earnest, active, conscientious workers, endeavoring to benefit humanity—a noble work that involves the freedom and liberty of the present and future generations.

Considered from a physical, moral and social standpoint in the present day and generation, we are constrained to say all men are born unequal.

We as a nation and people are doing things every day, not essential to life and happiness, which are but links in the iron chain that clog the advancement and progress of our children and the next generation.

In the name of liberty purchased by the sufferings and blood of our forefathers we indulge in vices and immorality, producing disease and degeneration of ourselves, and hand them down as precious heirlooms to the next generation.

In the name of liberty we allow the criminal, the degenerate, and the diseased, to marry and propagate their kind.

Yea, even in the name of liberty at times we sanction and license, by the majesty of the law, the very things that produce crime, degeneracy and disease.

It is time we were organizing for a determined, persistent, mighty effort to establish a higher standard of citizenship based upon the proper conception of liberty and freedom, and not license.

Absolute liberty requires absolute purity and the practical application of the golden rule.

He who indulges in vice and immorality and habitually violates physical laws not only binds himself, but those coming after him, by the laws of heredity and acquired characteristics.

May we at this time inaugurate a movement that will break the iron chain of crime, vice and disease, that binds so many of our fellow beings and substitute that golden chain that will lead them onward and upward to higher and nobler lives, whose ultimate goal is absolute freedom, where all men are really and truly born equal.

